# Bisphenol A Promotes Cell Survival Following Oxidative DNA Damage in Mouse Fibroblasts

**DOI:** 10.1371/journal.pone.0118819

**Published:** 2015-02-18

**Authors:** Natalie R. Gassman, Erdem Coskun, Donna F. Stefanick, Julie K. Horton, Pawel Jaruga, Miral Dizdaroglu, Samuel H. Wilson

**Affiliations:** 1 Genomic Integrity and Structural Biology Laboratory, NIEHS, National Institutes of Health, 111 T.W. Alexander Drive, Research Triangle Park, NC 27709, United States of America; 2 Biomolecular Measurement Division, National Institute of Standards and Technology, Gaithersburg, MD 20899, United States of America; 3 Faculty of Pharmacy, Gazi University, Ankara, Turkey; University of South Alabama Mitchell Cancer Institute, UNITED STATES

## Abstract

Bisphenol A (BPA) is a biologically active industrial chemical used in production of consumer products. BPA has become a target of intense public scrutiny following concerns about its association with human diseases such as obesity, diabetes, reproductive disorders, and cancer. Recent studies link BPA with the generation of reactive oxygen species, and base excision repair (BER) is responsible for removing oxidatively induced DNA lesions. Yet, the relationship between BPA and BER has yet to be examined. Further, the ubiquitous nature of BPA allows continuous exposure of the human genome concurrent with the normal endogenous and exogenous insults to the genome, and this co-exposure may impact the DNA damage response and repair. To determine the effect of BPA exposure on base excision repair of oxidatively induced DNA damage, cells compromised in double-strand break repair were treated with BPA alone or co-exposed with either potassium bromate (KBrO_3_) or laser irradiation as oxidative damaging agents. In experiments with KBrO_3_, co-treatment with BPA partially reversed the KBrO_3_-induced cytotoxicity observed in these cells, and this was coincident with an increase in guanine base lesions in genomic DNA. The improvement in cell survival and the increase in oxidatively induced DNA base lesions were reminiscent of previous results with alkyl adenine DNA glycosylase-deficient cells, suggesting that BPA may prevent initiation of repair of oxidized base lesions. With laser irradiation-induced DNA damage, treatment with BPA suppressed DNA repair as revealed by several indicators. These results are consistent with the hypothesis that BPA can induce a suppression of oxidized base lesion DNA repair by the base excision repair pathway.

## Introduction

Bisphenol A (BPA) is found in a variety of consumer products such as adhesives, food and beverage containers, and dental composites and sealants [1]. Concern about BPA exposure is often linked to its estrogenic properties, but the affinity of BPA for cellular estrogen receptors is much lower than that of estradiol [2,3]. Additionally, there are inconsistent data regarding genotoxicity of BPA [4–7]. Despite these inconsistencies, BPA exposure has been shown to cause DNA damage independently of its estrogenic properties [2,6,8–10]. The response of DNA repair pathways to BPA exposure and BPA-induced DNA damage, however, has not been extensively investigated.

DNA damaging effects of BPA are thought to occur indirectly through the generation of reactive oxygen species (ROS). ROS create stable base lesions and abasic sites in genomic DNA [11–14]. While previous studies had pointed to DNA damaging effects of BPA, the oxidatively induced DNA damage produced by BPA exposure has not been investigated, nor has BPA exposure in combination with other DNA damaging agents, especially other oxidizing agents. The ubiquity of BPA results in exposure concurrent with endogenous and exogenous DNA damaging events, like oxidative stress or environmental toxicants, and together these exposures can increase the DNA damage load of genomic DNA and have implications for genomic stability and human disease development and progression. In the current study, we sought to address the influence of BPA on the oxidative DNA damage response in the model experimental system of cultured mouse fibroblasts.

The base excision repair (BER) pathway is the main repair system responsible for removal of modified bases (such as 8-oxo-guanine (8-oxoGua) and 2,6-diamino-4-hydroxy-5-formamidopyrimidine (FapyGua)) formed upon oxidative stress. In the cases of the 8-oxoGua and FapyGua lesions, 8-oxoGua-DNA glycosylase (OGG1) removes the lesions from double-stranded genomic DNA leaving abasic sites. While OGG1 is known as a bifunctional enzyme capable of carrying out both base removal and AP-lyase activity, cleaving the phosphodiester bond of the resulting abasic site by a β- or β-δ-elimination mechanism, its AP-lyase activity is relatively weak [15–17]. Therefore, another enzyme, AP endonuclease 1 (APE1) incises the abasic site, resulting in a single-nucleotide gapped DNA with 3´-OH and 5´-dRP groups at the gap margins. Subsequently, DNA polymerase β (Pol β) loads onto this BER intermediate, removes the 5´-dRP group, and then fills the single-nucleotide gap. DNA ligase I, or in some cases the ligase III α-XRCC1 complex, then seals the nick in the repair intermediate to complete the pathway. Repair of other oxidized bases, such as 5-hydroxycytosine (5-OH-Cyt), thymine glycol (ThyGly), and 4,6-diamino-5-formamidopyrimidine (FapyAde), are initiated by other DNA glycosylases, e.g., NEIL1 and NTH, and these glycosylases have overlapping substrate specificities including excision of FapyGua by NEIL1 [18,19]. Cells make use of the BER pathway as a first-line defense against oxidized base damage induced by endogenous and exogenous agents, but other DNA repair pathways can back-up a deficiency in BER.

To examine an effect of BPA on the response to oxidative stress in mouse fibroblasts, we chose to use a Ku70-deficient cell line. These cells were selected because they are deficient in strand break repair by non-homologous end joining (NHEJ), a back-up repair pathway for BER [20,21]. Thus, NHEJ-deficient cells provide an opportunity for study of BER responses to oxidatively induced DNA damage in the absence of a back-up repair pathway. Cell phenotypes were characterized after treatment with the oxidative stress agent KBrO_3_, or with the combination of BPA plus KBrO_3_.

## Material and Methods

### Cell culture

Ku70^+/+^ and Ku70^-/-^ (a gift from Dr. Shigemi Matsuyama, Cleveland, OH) mouse embryonic fibroblasts (MEFs) were grown at 37°C in a 10% CO_2_ incubator in Dulbecco’s modified Eagle’s medium (DMEM) supplemented with glutamine, 10% fetal bovine serum (FBS; HyClone, Logan, UT), 1% non-essential amino acids, and 1% sodium pyruvate [22]. Ogg1^+/+^ and Ogg1^-/-^ MEFs (a gift from Dr. Arne Klungland, Oslo, Norway) were grown as above in DMEM supplemented with GlutaMAX-1 (Life Technologies, Carlsbad, CA) and 10% FBS. Cells were routinely tested and found to be free of mycoplasma contamination.

### Cytotoxicity studies

Cytotoxicity was determined by growth inhibition assays. We consider this cell survival assay to be more reliable in MEFs than alternate assays such as clonogenic colony counting or short-term cell killing assays. Results obtained with the cell survival assay have been confirmed using other assays. Cells were seeded at a density of 40,000 cells/well for Ku70^+/+^ and Ku70^-/-^ and 50,000 cells/well for Ogg1^+/+^ and Ogg1^-/-^ in six-well dishes. The following day, cells were exposed to a range of concentrations of BPA alone for 24 h or KBrO_3_ alone for 1 h. In other cases, cells were exposed to 150 μM BPA for 1 h, then a range of KBrO_3_ concentrations for 1 h, and finally with 150 μM BPA for a further 23 h. BPA and KBrO_3_ were from Sigma-Aldrich. BPA was prepared in absolute ethanol and diluted to the final working concentrations in medium. KBrO_3_ was dissolved directly in the medium at the time of the experiment. For KBrO_3_ alone and BPA plus KBrO_3_ co-exposures, after the 1 h KBrO_3_ treatment, the cells were washed with Hanks’ balanced salt solution (HBSS) and fresh medium was added with or without BPA. After 24 h exposure to BPA, cells were washed in HBSS and fresh medium was added. Dishes were then incubated for 6–7 days at 37°C in a 10% CO_2_ incubator until untreated control cells were approximately 80% confluent. Cells (triplicate wells for each drug concentration) were counted by a cell lysis procedure [23], and results were expressed as the number of cells in drug-treated wells relative to cells in control wells (% control growth).

### Measurement of intracellular ROS

The level of intracellular ROS was measured by CM-H_2_DCFDA (Life Technologies) similar to [24]. Ku70^-/-^ were seeded in 100 mm dishes at 5×10^5^ cells/dish and treated as described. 1 h after KBrO_3_ exposure, the cells were harvested using 0.25% trypsin. A 1 mM stock of CM-H_2_DCFDA in anhydrous DMSO (Sigma-Aldrich) was prepared and diluted to 5 μM in PBS. After centrifuging and washing cells with PBS, cell pellets were resuspended in the 5 μM CM-H_2_DCFDA and incubated at 37°C for 60 min in the dark. Stained cells were analyzed with Becton Dickinson LSRII flow cytometer (BD, Franklin Lakes, NJ, USA), and the mean fluorescent intensity was recorded.

### Isolation of nuclear DNA

Cell were treated as described above and allowed to repair for 4 h. After this period, the cells were washed twice in ice-cold phosphate-buffered saline (PBS) and harvested. DNA was isolated as previously described [25]. Briefly, cell pellets were resuspended in 2 mL of lysis buffer (10 mM Tris-HCl, pH 8.2, 2 mM EDTA, 0.4 M NaCl, and 1% SDS) with 2 mg/mL of proteinase K and incubated for 18 h at 37°C. A ¼ volume of saturated NaCl solution was added to the lysis buffer, and the mixture was vortexed well, then centrifuged at 12000 x g for 15 min at 4°C. Cold ethanol (96%) was added to the supernatant fraction in a ratio of 2.5 to 1. The mixture was kept at -20°C for 18 h. DNA pellets were separated and resuspended in 1 ml of TE buffer (Sigma Aldrich). RNase A was added to a final concentration of 0.2 mg/mL and the mixture was incubated at 37°C for 1 h. An equal volume of lysis buffer with proteinase K was added, and the mixture was incubated for 1 h at 55°C. A ¼ volume of saturated NaCl solution was added and the mixture was vortexed well, then centrifuged at 12000 x g for 15 min at 4°C. DNA was precipitated from the supernatant fraction with cold ethanol (96%) at -20°C for 18 h. The supernatant fraction and DNA pellet were then separated by centrifugation, and the DNA pellet was washed several times with 70% ethanol, and then dried under vacuum.

### Gas chromatography/tandem mass spectrometry

Gas chromatography/tandem mass spectrometry (GC-MS/MS) with isotope-dilution was used to identify and quantify modified DNA bases in DNA. Isolated DNA samples were dissolved in water at 4°C overnight. The UV spectrum of each DNA sample was recorded by absorption spectrophotometry between the wavelengths of 200 nm and 350 nm to ascertain the quality of DNA and an accurate quantification of the DNA concentration. The absorbance at 260 nm was used to measure the DNA concentration of each sample (absorbance of 1 = 50 μg of DNA/mL). Aliquots (50 μg) of DNA samples were dried in a SpeedVac under vacuum. Subsequently, aliquots of 5-OH-Cyt-^13^C,^15^N_2_, ThyGly-d_4_, FapyAde-^13^C,^15^N_2_, FapyGua-^13^C,^15^N_2_ and 8-oxo-Gua-^15^N_5_ were added as internal standards. DNA samples were dissolved in 50 μL of an incubation buffer consisting of 50 mM phosphate buffer (pH 7.4), 100 mM KCl, 1 mM EDTA, and 0.1 mM dithiothreitol, and then incubated with 2 μg of *E. coli* Fpg and 2 μg of *E. coli* Nth for 1 h at 37°C to release 5-OH-Cyt, ThyGly, FapyAde, FapyGua and 8-oxo-Gua from DNA. Subsequently, 100 μL ethanol were added to precipitate DNA. After centrifugation, supernatant fractions were separated, lyophilized and trimethylsilylated. Derivatized samples were analyzed by GC-MS/MS as described previously [26].

### Micro-irradiation and immunofluorescence

Wild-type and Ku70-deficient cells were seeded at 2 × 10^5^ cells per dish on 35 mm glass bottomed petri dishes containing an etched grid (MatTek, Ashland, MA) and incubated in growth medium supplemented with 10 μM BrdU (Sigma-Aldrich). After 24 h, the BrdU was removed, and when indicated, 150 μM BPA was added, and cells were incubated for 1 h prior to irradiation. Samples were then imaged using a 40x C-Apochromat (numerical aperture 1.2) water immersion objective coupled to a Zeiss LSM510 META confocal microscope (Carl Zeiss MicroImaging). Base lesions and strand breaks were introduced by UV laser micro-irradiation at 364 nm (Coherent Enterprise II) with intensities equivalent to 0.176 μJ as described previously [27]. After micro-irradiation, cells were either immediately fixed in 4% paraformaldehyde or allowed to recover in a 37°C incubator for the times indicated. After fixation, cells were permeabilized with 0.25% Triton X-100 in PBS for 10 min, washed three times in PBS, then further permeabilized and blocked with PBS + 1% BSA for 30 min. Cells were then incubated with anti-XRCC1 antibody (1:50; Abcam, Cambridge, MA) and anti-PAR antibody (1:100; Abcam) for 1 h. Cells were washed three times with PBS, then incubated in Alexa 488 conjugated anti-mouse and Alexa 647 conjugated anti-chicken antibody (1:2,000; Life Technologies) for 1 h. Fluorescence images were acquired with the same 40x water immersion objective on the LSM510. Recruitment of XRCC1 and synthesis of PAR at the site of DNA damage were measured using IMAGEJ. The mean intensity of the irradiated site was determined as previously described [27]. Values of 0 for mean intensity reflect equal distribution of proteins throughout the nucleus, while values above 0 reflect an increasing protein concentration along the site of DNA damage. Each experiment was repeated on at least twenty cells, and the data presented represent mean values. Images are representative.

γH2AX foci were detected in untreated and treated Ku70 wild type and deficient cells and Ogg1 wild type and deficient cells. Briefly, cells were plated on coverslips at 2 × 10^5^ cells/coverslip. The following day cells were either untreated or treated with 150 μM BPA, 20 mM KBrO_3_, or both BPA and KBrO_3_ as described above. Cells were then washed in HBSS, and fresh medium was added with or without BPA for an additional 3 h repair time. After the indicated repair time, cells were fixed in 4% paraformaldehyde, then permeabilized with 0.25% Triton X-100 in PBS for 10 min, washed three times in PBS, then further permeabilized and blocked with PBS + 1% BSA for 30 min. Cells were then incubated with anti-γH2AX antibody (1:200; Millipore) for 1 h. Cells were washed three times with PBS and then incubated in Alexa 488 conjugated anti-mouse antibody for 1 h. Cells were then mounted on coverslides using ProLong Gold with DAPI (Life Technologies). Fluorescence images were acquired with the same 40x water immersion objective on the LSM510, and the foci were detected using MetaMorph software. Each experiment was repeated on at least hundred cells, and the data presented represent mean values.

### Statistical analyses

All values were expressed as mean ± standard error of mean (SEM), except quantifications of modified DNA bases are expressed as mean ± standard deviation (SD). The data were analyzed by means of one-way analysis of variance (ANOVA) and Student’s t-test. p < 0.05 denoted by * were considered to correspond with statistical significance.

## Results

As noted above, a mouse fibroblast cell line with a deletion in the Ku70 gene was selected for study along with the paired wild-type cells. No difference in BPA-induced cytotoxicity was observed between these two cell lines, and a minimally cytotoxic dose of 150 μM BPA was chosen for the studies to be described. It is important to note that this level of BPA did not increase cellular proliferation (S1 Fig.).

### Co-treatment with BPA decreases KBrO_3_-induced cytotoxicity

**Fig 1:**
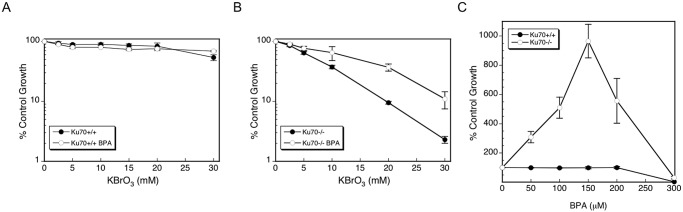
Cell survival following co-exposure of BPA and KBrO_3_. Ku70-proficient (A) and-deficient (B) cells were treated for 1 h with the oxidizing agent, KBrO_3_, with and without pre-treatment with 150 μM BPA for 1 h prior to induction of KBrO_3_-induced DNA damage. Exposure to BPA was for a total of 24 h (see Materials and Methods). (C) Dose of BPA required for maximal protective effect. For determination of the dose of BPA that produces the maximal protective effect against 20 mM KBrO_3_, Ku70-proficient and-deficient cells were treated with increasing doses of BPA for 1 h, co-exposed with KBrO_3_ for 1 h, and then the BPA exposure was continued for a total of 24 h (see Materials and Methods). The results are expressed as the number of cells in BPA-treated wells relative to number of cells in wells treated with KBrO_3_ alone.

The Ku70-deficient cells used here were previously shown to be sensitive to oxidative DNA damage [21]. During the initial characterization of wild-type and Ku70-deficient cells with the oxidative damaging agent KBrO_3_, wild-type cells were fairly resistant to KBrO_3_ and co-treatment with BPA had no effect (Fig. 1A). In contrast, the Ku70-deficient cells were sensitive to KBrO_3_, and interestingly, these cells showed an increase in cell viability with BPA co-treatment (Fig. 1B). The BPA protective effect was observed over a range of KBrO_3_ concentrations from 5 to 30 mM (Fig. 1B). The maximal BPA protective effect against 20 mM KBrO_3_-induced cell killing was at 150 μM BPA (Fig. 1C).

A mechanism that could account for the observed protective effect was suggested in previous studies of alkylation base damage and cells with a deficiency in alkyladenine DNA glycosylase (AAG) [28]. A protective effect against alkylating agent-induced cytotoxicity was observed upon deletion of the glycosylase gene, and this was considered to reflect a reduction in toxic DNA strand break intermediates of BER, whereas the unrepaired alkylated bases themselves are not cytotoxic [28,29]. Next, we investigated the possibility of a similar mechanism for the BPA protective effect against oxidative stress observed in Fig. 1.

**Fig 2:**
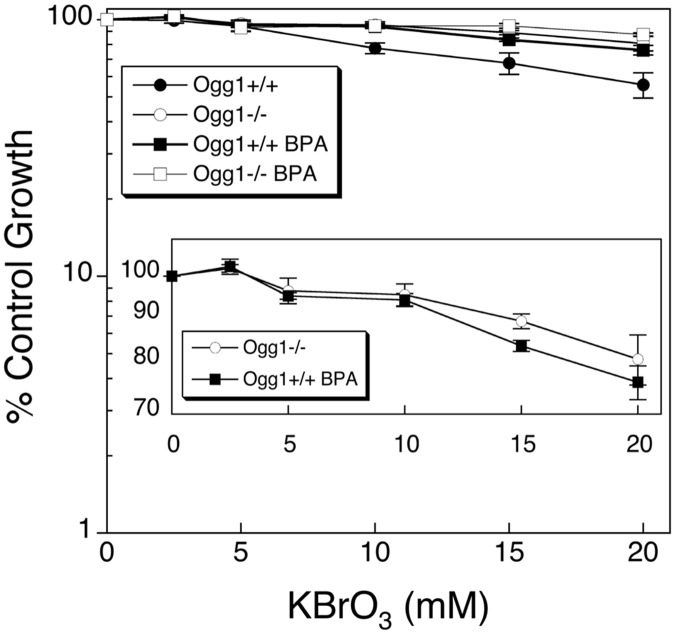
Lack of a BPA co-exposure protective effect in Ogg1-deficient cells. Ogg1-proficient and deficient cells were treated for 1 h with the oxidizing agent KBrO_3_ with and without pre-treatment with 150 μM BPA for 1 h prior to the induction of KBrO_3_-induced DNA damage and then for a total of 24 h (see Materials and Methods). Inset, comparison of Ogg1-proficient cells co-exposed to BPA and KBrO_3_ and the Ogg1-deficient cell line exposed to KBrO_3_ alone.

Oxidative damaging agents like KBrO_3_ generate several types of DNA lesions, with 8-oxoGua and FapyGua among the most abundant. These lesions are excised by the DNA glycosylase Ogg1 and the protective effect of BPA could be due to a reduction in Ogg1 removal of oxidized base damage [28,29]. To examine this idea, paired Ogg1-proficient and-deficient cell lines were treated with KBrO_3_ either alone or in co-treatment with BPA. As shown in Fig. 2, the Ogg1-proficient cells were modestly sensitive to KBrO_3_ at the highest levels tested, and co-treatment with BPA protected these cells from KBrO_3_-induced cytotoxicity. In contrast, the Ogg1-deficient cells were resistant to KBrO_3_-induced cytotoxicity over this same concentration range, and a BPA protective effect was not observed (Fig. 2). This would be expected if BPA suppression of Ogg1-initiated repair were responsible for the protective effect. Further, if BPA suppresses Ogg1 activity, then KBrO_3_ sensitivity in BPA co-treated wild-type cells and Ogg1-deficient cells would be similar, as confirmed in the experiments shown in Fig. 2.

### BPA exposure increases intracellular ROS

**Fig 3:**
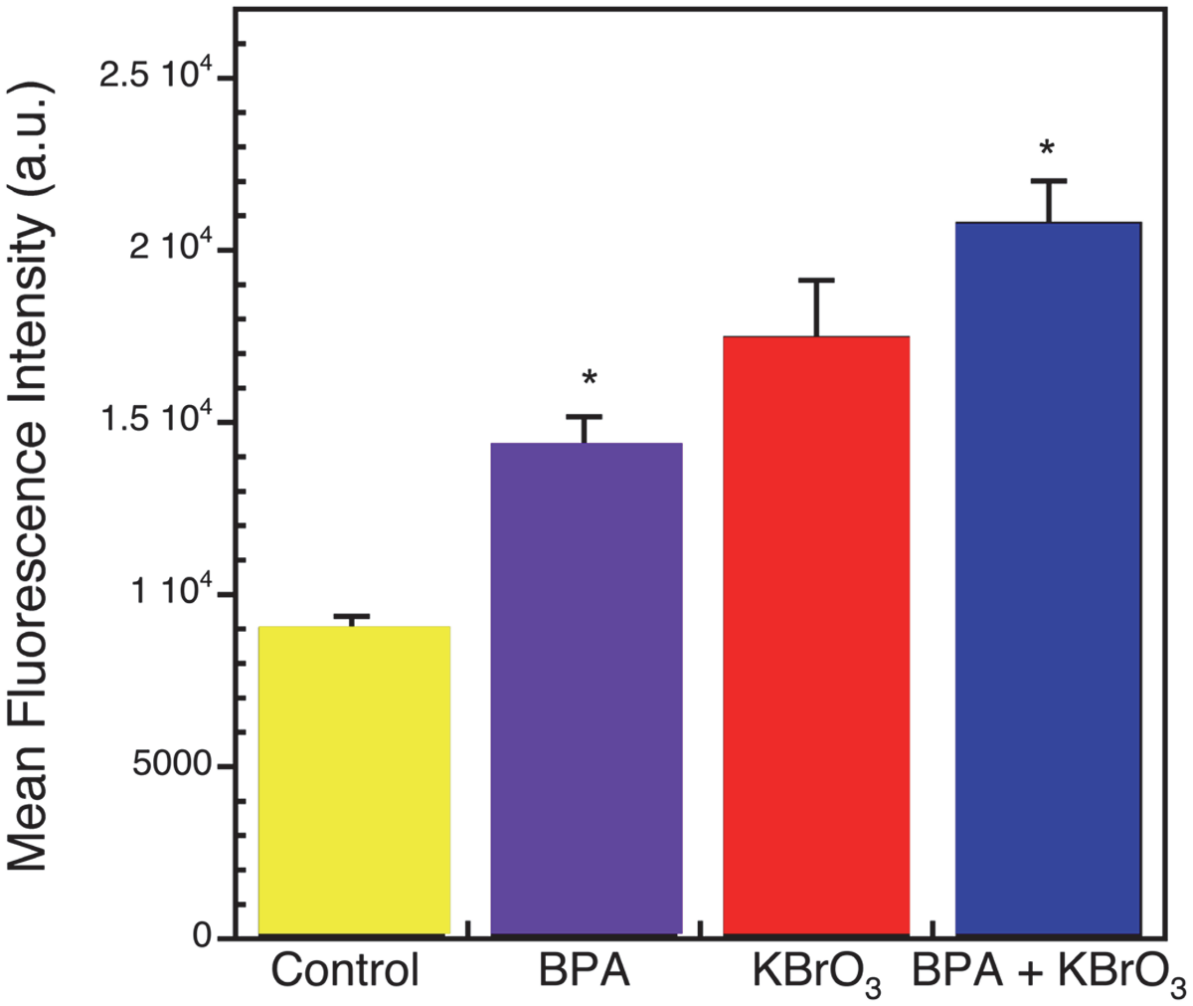
Effect of BPA on intracellular ROS production. Ku70-deficient cells were treated for 1 h with KBrO_3_ with and without pre-treatment with 150 μM BPA for 1 h prior to induction of KBrO_3_-induced DNA damage. After KBrO_3_ exposure, 5 μM CM-H_2_DCFDA was added to the cells and incubation was continued at 37°C for 60 min in the dark. Stained cells were analyzed by flow cytometery and mean fluorescent intensity was recorded. Each bar represents mean ± SEM of three independent experiments (*p < 0.05 vs. control).

To verify that ROS were being generated by the exposures in Fig. 1, we determined the levels of intracellular ROS induced by the treatments. CM-H_2_DCFDA fluorescent dye was used to evaluate the generation of ROS in untreated Ku70-deficient cells and 1 h after treatment with BPA, KBrO_3_, or co-treatment with BPA and KBrO_3_ (Fig. 3). A significant increase of the fluorescence intensity over the control was observed after exposure to BPA (p = 0.002) and co-exposure to BPA and KBrO_3_ (p = 0.0012), and a slightly significant increase was observed after KBrO_3_ alone (p = 0.055, Fig. 3).

### Co-treatment with BPA and KBrO_3_ generates oxidatively induced DNA lesions

**Fig 4:**
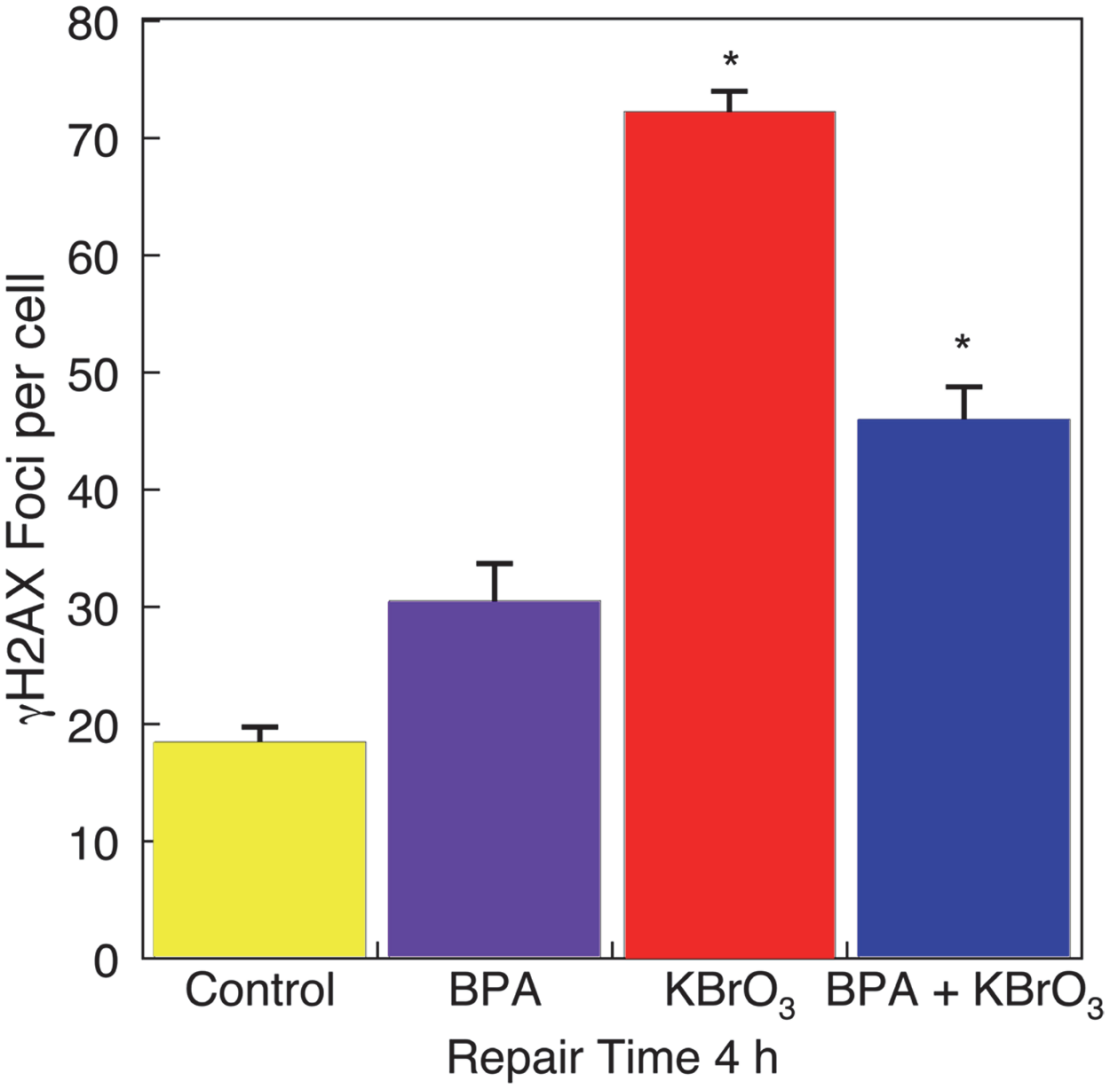
Strand break signaling by γH2AX in Ku70-deficient cells. **γ**H2AX foci were measured at 4 h after exposure to BPA alone, KBrO_3_ alone, and after co-exposure (see Material and Methods). Each bar represents mean ± SEM of three independent experiments (*p < 0.05 vs. control).

To determine if BPA and KBrO_3_ co-treatment altered the levels of oxidatively induced DNA base lesions, we quantified five oxidatively induced DNA lesions in DNA isolated from Ku70-deficient cells treated with BPA alone, KBrO_3_ alone_,_ or co-treated with BPA and KBrO_3_ (Table 1). GC-MS/MS with isotope-dilution, as described [26], was used with nuclear DNA isolated from untreated control cells or treated cells 4 h after treatment. The results are shown in Table 1 and summarized in Fig. 4.

**Table 1:** Levels of selected modified DNA bases in genomic DNA in Ku70-/-.

	DNA lesion/ 10^6^ DNA bases (mean ± SD, n ≥3)				
	Ku70^-/-^				
	5-OH-Cyt	ThyGly	FapyAde	FapyGua	8-oxoGua
Control	2.10± 0.64	3.82± 1.32	2.83± 0.98	3.69± 1.41	0.98 ± 0.16
BPA	4.74± 1.16*	8.41± 0.45*	3.72± 1.37	5.19 ± 0.92	1.21 ± 0.4
KBrO_3_	5.48± 0.89 *	5.11± 0.69	4.01± 0.86	4.6 ± 0.92	1.33 ± 0.55
BPA + KBrO_3_	4.08± 0.48*	7.48± 0.48*	4.38±0.41*	6.77 ± 1.36*	1.55 ± 0.59

Means of at least three replicates are shown (*p < 0.05 vs. control).

As shown in Table 1, treatment with BPA alone generated DNA lesions, consistent with the increase in ROS observed in Fig. 3; BPA alone significantly increased the levels of 5-OH-Cyt and ThyGly over control (p = 0.018 and p = 0.019, respectively). Small, but non-significant, increases in the levels of FapyAde, FapyGua and 8-oxo-Gua were also observed. Treatment with KBrO_3_ alone also resulted in a significant increase in the level of 5-OH-Cyt over control (p = 0.018) and slight, but non-significant, increases in the levels of the other DNA lesions were observed. Co-treatment with BPA and KBrO_3_ significantly increased the levels of 5-OH-Cyt (p = 0.05), ThyGly (p = 0.046), FapyAde (p = 0.045) and FapyGua (p = 0.016) lesions over control. These results demonstrate that the treatment with BPA generated oxidatively induced DNA lesions that are persistent in the DNA and that co-treatment with KBrO_3_ significantly increased the level of FapyGua base lesions in genomic DNA. For comparison, these five lesions also were measured in DNA from Ku70^+/+^ cells (S1 Table). Ku70^+/+^ cells showed a lower modified DNA base content than Ku70^-/-^ cells, though a significant increase in FapyGua base lesions was still observed after co-treatment.

### Co-treatment with BPA and KBrO_3_ reduces strand break signaling

If BPA suppresses repair of KBrO_3_-induced 8-oxoGua and FapyGua lesions, then strand break intermediates of BER may be decreased in cells co-treated with BPA compared with KBrO_3_ alone. To evaluate this possibility, we examined strand break signaling in Ku70-deficient cells using an immunoassay for γH2AX production. Cells were co-treated with BPA and KBrO_3_ and then cultured for 4 h. Cells were then fixed and γH2AX foci per cell were quantified. After BPA and KBrO_3_ co-treatment, the number of γH2AX foci was lower than that with KBrO_3_ treatment alone (Fig. 4). This result is consistent with the lack of foci change observed in Ogg1-/- cells after treatment with KBrO_3_ (S2 Fig.). These results are consistent with the idea that BPA suppressed repair of the KBrO_3_-induced lesions and the strand break intermediates of repair.

Strand break assessment was also conducted using the single-cell comet assay (not shown). Although there appeared to be a modest reduction in strand breaks with BPA and KBrO_3_ co-treatment, the results with this assay failed to yield significant differences as a function of the various treatments. The difference in results with these two strand break assays could be due to a difference in sensitivity.

### Co-treatment with BPA and laser-delivered micro-irradiation

**Fig 5:**
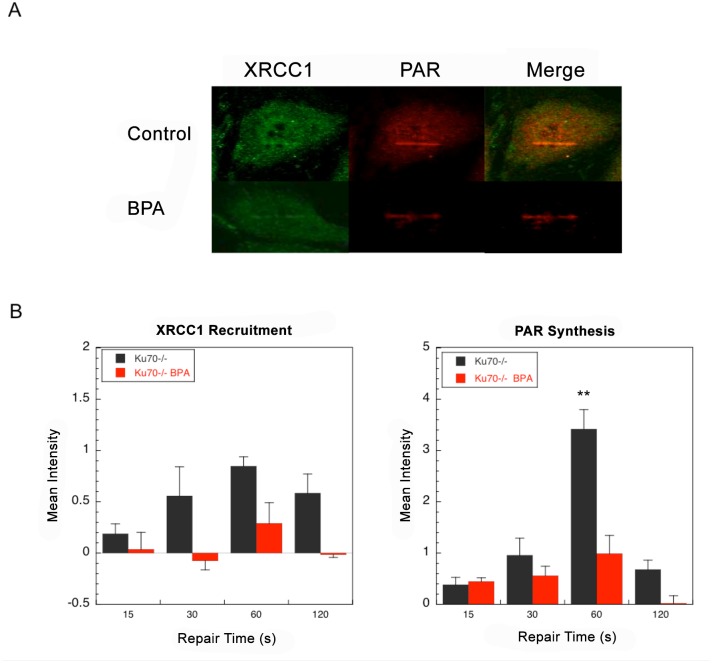
Recruitment of BER factors to sites of laser-induced DNA damage in Ku70-deficient cells. Recruitment of XRCC1 and PAR polymer accumulation were measured at sites of DNA damage in Ku70-deficient cells as described in Materials and Methods. (A) Images obtained one minute after irradiation of Ku70-deficient cells with and without 150 μM BPA. (B) Time courses of XRCC1 recruitment and PAR synthesis to the site of damage in Ku70-deficient cells with the repair times specified. Images are representative; at least 15 cells were measured for every time point and each bar represents mean ± SEM of three independent experiments (*p < 0.05 vs. control). Scale bars are 20 μM.

To further investigate the effect of BPA on base lesion repair, laser-delivered micro-irradiation was utilized to produce DNA damage. Then, recruitment of the BER protein XRCC1 and the synthesis of poly(ADP-ribose) (PAR) by PARP-1 at the sites of laser-delivered damage were measured [27,30,31]. The DNA repair status of cells was assessed by monitoring the intensity of these signals as a function of time [27,30,31]. Without BPA treatment, recruitment of XRCC1 and accumulation of PAR were observed within seconds after irradiation and reached the highest levels at 60 s post-irradiation (Fig. 5). Interestingly, in the presence of BPA, recruitment of XRCC1 was suppressed and PAR accumulation was strongly reduced (Fig. 5B). These results are consistent with a BPA-induced suppression of DNA repair after laser-delivered DNA damage.

## Discussion

Multiple overlapping repair pathways characterize the process of cellular DNA repair. There are many examples where repair of the same DNA lesion occurs simultaneously by BER, mismatch repair, double-strand break repair and nucleotide excision repair. In studies in yeast, for example, recombination and double-strand break repair are the dominant repair pathways for methylated base lesions. Thus, various repair pathways other than BER can conduct base lesion repair. In the present study, Ku70-deficient mouse fibroblasts were chosen to encourage repair of KBrO_3_-induced damage by the BER pathway. Elimination of the NHEJ back-up pathway revealed a BPA deregulation of repair that was largely masked in the isogenic wild-type cells. We also chose to examine the effects of high doses of BPA during a relatively short-term period to concentrate the effects of BPA. Further studies will be required to examine BPA effects upon long-term exposure or at lower levels of BPA.

In the Ku70-deficient cell line, the cytotoxicity induced by KBrO_3_ was partially reversed by BPA co-treatment. The maximal BPA protective effect on cell survival using an intermediate level of KBrO_3_ (20 mM) was observed at 150 μM BPA (Fig. 1C). This level of BPA exposure is much higher than that anticipated for environmental exposures, but is similar to that used in other laboratory studies [2,24,32–34]. As noted above, the BPA protective effect is reminiscent of effects previously observed for AAG-deficient cell lines treated with alkylating agents [28,29]. In line with this, an Ogg1-deficient cell line was not sensitive to KBrO_3_, even at higher concentrations (Fig. 2). The Ogg1-proficient cell line showed sensitivity to increasing concentrations of KBrO_3_. This sensitivity was stronger than that observed in the Ku70-proficient cell line; however, repair capacities can vary considerably between non-isogenic cell lines due to a number of factors, i.e., the isolation conditions, immortalization procedures, medium and growth conditions used. Co-exposure with BPA ameliorated the sensitivity of both wild-type cells though the effect of the more sensitive Ogg1-proficient cells is more pronounced (Fig. 2, inset). This is consistent with the idea that toxic DNA repair strand break intermediates were suppressed by BPA and that the lesions created by KBrO_3_ were not cytotoxic.

To verify that ROS and DNA lesions were induced by the BPA and KBrO_3_ treatments used here, we determined the levels of intracellular ROS produced by the three treatment conditions and quantified oxidatively induced lesions in genomic DNA by mass spectrometry. Examination of DNA from the Ku70-deficient cells after treatment with BPA revealed a significant increase in the levels of 5-OH-Cyt and ThyGly, and co-treatment with KBrO_3_ significantly generated all lesions except for 8-oxo-Gua (Table 1). This increase in lesion load was consistent with a reduction in DNA repair after BPA exposure. Previous studies showed significant increases in the levels of FapyAde and FapyGua in *neil1* ^*-/-*^ mice, and in the levels of FapyGua and 8-oxoGua in *ogg1* ^*-/-*^ mice [35–37]. The changes in levels observed in those studies are consistent with increases observed here after BPA co-treatment, though a significant increase in the level of 8-oxoGua was observed in *ogg1* ^*-/-*^ mice [37].

A reduction in lesion removal may be expected to correlate with a decrease in DNA damage signaling by γH2AX [38]. Examination of γH2AX foci after co-treatment with BPA revealed a significant reduction in DNA damage signaling. An attempt was made to confirm a decrease in strand breaks by the comet assay, but the differences observed were not large enough to provide a statistically significant result. These observations do suggest that co-treatment with BPA altered the initiation of DNA repair of KBrO_3_-induced lesions.

We next used laser-delivered micro-irradiation to create DNA lesions and strand breaks. This DNA damaging technique makes use of a calibrated dose of laser energy to create a mixture of single strand breaks and abasic sites along with abundant oxidatively induced base damage [27,30,31,39,40]. Previous work had established that the method can be used to monitor recruitment of BER factors, like XRCC1 and Pol β, to the damage site and that the amount and residence time of PAR polymers at the damage site is as an indicator of the repair process [27,30,31,40]. Interestingly, with BPA co-treatment of Ku70-deficient cells, PAR accumulation was reduced, and XRCC1 recruitment was negligible (Fig. 5). These results were consistent with the idea that DNA repair initiation is suppressed by BPA. Finally, the reduction in PAR level observed here after micro-irradiation indicates that PARP-1 activation was suppressed at the DNA damage sites. A reduction in DNA strand breaks would suppress activation of PARP-1, as well as recruitment of a DNA repair factors like XRCC1 (Fig. 5).

The results described here identify a BPA-dependent protective effect against the cytotoxicity of KBrO_3_-induced oxidative stress. This improvement in cell survival after co-exposure may be compared with several previous studies noting protective effects with co-exposure to BPA and other DNA damaging agents. Dobrzynska et al. found that co-exposure of BPA with X-rays reduced DNA strand breaks compared to single agent treatment. The mechanism for this reduction was not determined, but it was speculated that combined exposure prevented strand breaks [41]. A study by Nishimura et al. noted protection against oxidative injury with BPA exposure for 30–45 days and then co-exposure with H_2_O_2_ [8]. The authors attributed enhanced cell survival to alterations in expression levels of GPER30 induced by epigenetic pathways and decreases in histone H3 methylation [8]. Taken together, these studies indicate several potential mechanisms for improved cell survival by BPA co-exposure. The short exposure window of the Dobrzynska et al. study and the reduction in strand breaks they observed is consistent with suppression of initiation of DNA repair. Further studies at longer intervals after treatment are necessary to determine if gene expression level changes and epigenetic pathways also play a role in long-term survival after genotoxic insult. However, our current results support an immediate effect of BPA in suppression of the initiation of DNA repair, leading to improved cell survival and a reduction in toxic DNA repair intermediates.

## Supporting Information

S1 Fig Cell proliferation measured 48 h after exposure to BPA. Cells were exposed to 150 μM BPA for 24 h as described in Material and Methods. Cells were then washed, normal growth medium was replaced, and cell survival was evaluated by CellTiter 96 AQueous One Solution Cell Proliferation Assay (Promega). Values shown are the percent change in cell number compared with control given as the mean ± SD of three independent experiments.
(TIF)

S2 Fig Strand break signaling by γH2AX in Ogg1—proficient and—deficient cells. **γ**H2AX foci were measured at 4 h after exposure to BPA alone, KBrO_3_ alone, and after co-exposure (see Material and Methods). Each bar represents mean ± SEM of three independent experiments.
(TIF)

S1 Table Levels of modified DNA bases in genomic DNA in Ku70^+/+^. (PDF)

## References

[1] Vandenberg LN, Hauser R, Marcus M, Olea N, Welshons WV (2007) Human exposure to bisphenol A (BPA). Reprod Toxicol 24: 139–177. 17825522 10.1016/j.reprotox.2007.07.010

[2] Iso T, Watanabe T, Iwamoto T, Shimamoto A, Furuichi Y (2006) DNA damage caused by bisphenol A and estradiol through estrogenic activity. Biol Pharm Bull 29: 206–210. 16462019 10.1248/bpb.29.206

[3] Lapensee EW, Tuttle TR, Fox SR, Ben-Jonathan N (2009) Bisphenol A at low nanomolar doses confers chemoresistance in estrogen receptor-alpha-positive and-negative breast cancer cells. Environ Health Perspect 117: 175–180. 10.1289/ehp.11788 19270784 PMC2649216

[4] Fernandez SV, Russo J (2010) Estrogen and xenoestrogens in breast cancer. Toxicol Pathol 38: 110–122. 10.1177/0192623309354108 19933552 PMC2907875

[5] Richter CA, Birnbaum LS, Farabollini F, Newbold RR, Rubin BS, et al (2007) In vivo effects of bisphenol A in laboratory rodent studies. Reprod Toxicol 24: 199–224. 17683900 10.1016/j.reprotox.2007.06.004 PMC2151845

[6] Tiwari D, Kamble J, Chilgunde S, Patil P, Maru G, et al (2012) Clastogenic and mutagenic effects of bisphenol A: an endocrine disruptor. Mutat Res 743: 83–90. 10.1016/j.mrgentox.2011.12.023 22245107

[7] vom Saal FS, Akingbemi BT, Belcher SM, Birnbaum LS, Crain DA, et al (2007) Chapel Hill bisphenol A expert panel consensus statement: integration of mechanisms, effects in animals and potential to impact human health at current levels of exposure. Reprod Toxicol 24: 131–138. 17768031 10.1016/j.reprotox.2007.07.005 PMC2967230

[8] Nishimura Y, Nakai Y, Tanaka A, Nagao T, Fukushima N (2014) Long-term exposure of 3T3 fibroblast cells to endocrine disruptors alters sensitivity to oxidative injury. Cell Biol Int 38: 868–874. 10.1002/cbin.10269 24604882

[9] Wu HJ, Liu C, Duan WX, Xu SC, He MD, et al (2013) Melatonin ameliorates bisphenol A-induced DNA damage in the germ cells of adult male rats. Mutat Res 752: 57–67. 10.1016/j.mrgentox.2013.01.005 23402883

[10] Yang YJ, Hong YC, Oh SY, Park MS, Kim H, et al (2009) Bisphenol A exposure is associated with oxidative stress and inflammation in postmenopausal women. Environ Res 109: 797–801. 10.1016/j.envres.2009.04.014 19464675

[11] Atkinson A, Roy D (1995) In vitro conversion of environmental estrogenic chemical bisphenol A to DNA binding metabolite(s). Biochem Biophys Res Commun 210: 424–433. 7755618 10.1006/bbrc.1995.1678

[12] Atkinson A, Roy D (1995) In vivo DNA adduct formation by bisphenol A. Environ Mol Mutagen 26: 60–66. 7641708 10.1002/em.2850260109

[13] Edmonds JS, Nomachi M, Terasaki M, Morita M, Skelton BW, et al (2004) The reaction of bisphenol A 3,4-quinone with DNA. Biochem Biophys Res Commun 319: 556–561. 15178442 10.1016/j.bbrc.2004.05.024

[14] Izzotti A, Kanitz S, D’Agostini F, Camoirano A, De Flora S (2009) Formation of adducts by bisphenol A, an endocrine disruptor, in DNA in vitro and in liver and mammary tissue of mice. Mutat Res 679: 28–32. 10.1016/j.mrgentox.2009.07.011 19660573

[15] Bjoras M, Luna L, Johnsen B, Hoff E, Haug T, et al (1997) Opposite base-dependent reactions of a human base excision repair enzyme on DNA containing 7,8-dihydro-8-oxoguanine and abasic sites. EMBO J 16: 6314–6322. 9321410 10.1093/emboj/16.20.6314 PMC1326315

[16] Rosenquist TA, Zharkov DO, Grollman AP (1997) Cloning and characterization of a mammalian 8-oxoguanine DNA glycosylase. Proc Natl Acad Sci U S A 94: 7429–7434. 9207108 10.1073/pnas.94.14.7429 PMC23838

[17] Roldan-Arjona T, Wei YF, Carter KC, Klungland A, Anselmino C, et al (1997) Molecular cloning and functional expression of a human cDNA encoding the antimutator enzyme 8-hydroxyguanine-DNA glycosylase. Proc Natl Acad Sci U S A 94: 8016–8020. 9223306 10.1073/pnas.94.15.8016 PMC21548

[18] Dizdaroglu M (2003) Substrate specificities and excision kinetics of DNA glycosylases involved in base-excision repair of oxidative DNA damage. Mutat Res 531: 109–126. 14637249 10.1016/j.mrfmmm.2003.07.003

[19] Hazra TK, Izumi T, Boldogh I, Imhoff B, Kow YW, et al (2002) Identification and characterization of a human DNA glycosylase for repair of modified bases in oxidatively damaged DNA. Proc Natl Acad Sci U S A 99: 3523–3528. 11904416 10.1073/pnas.062053799 PMC122556

[20] Choi YJ, Li H, Son MY, Wang XH, Fornsaglio JL, et al (2014) Deletion of Individual Ku Subunits in Mice Causes an NHEJ-Independent Phenotype Potentially by Altering Apurinic/Apyrimidinic Site Repair. PloS one 9: e86358 10.1371/journal.pone.0086358 24466051 PMC3900520

[21] Li H, Marple T, Hasty P (2013) Ku80-deleted cells are defective at base excision repair. Mutat Res 745–746: 16–25. 10.1016/j.mrfmmm.2013.03.010 23567907 PMC3721509

[22] Gama V, Gomez JA, Mayo LD, Jackson MW, Danielpour D, et al (2009) Hdm2 is a ubiquitin ligase of Ku70-Akt promotes cell survival by inhibiting Hdm2-dependent Ku70 destabilization. Cell Death Differ 16: 758–769. 10.1038/cdd.2009.6 19247369 PMC2669846

[23] Butler WB (1984) Preparing nuclei from cells in monolayer cultures suitable for counting and for following synchronized cells through the cell cycle. Anal Biochem 141: 70–73. 6496937 10.1016/0003-2697(84)90426-3

[24] Xin F, Jiang L, Liu X, Geng C, Wang W, et al (2014) Bisphenol A induces oxidative stress-associated DNA damage in INS-1 cells. Mutat Res Genet Toxicol Environ Mutagen 769: 29–33. 10.1016/j.mrgentox.2014.04.019 25344109

[25] Jaruga P, Kirkali G, Dizdaroglu M (2008) Measurement of formamidopyrimidines in DNA. Free Radic Biol Med 45: 1601–1609. 10.1016/j.freeradbiomed.2008.09.019 18926902

[26] Reddy PT, Jaruga P, Kirkali G, Tuna G, Nelson BC, et al (2013) Identification and quantification of human DNA repair protein NEIL1 by liquid chromatography/isotope-dilution tandem mass spectrometry. J Proteome Res 12: 1049–1061. 10.1021/pr301037t 23268652

[27] Gassman NR, Stefanick DF, Kedar PS, Horton JK, Wilson SH (2012) Hyperactivation of PARP triggers nonhomologous end-joining in repair-deficient mouse fibroblasts. PloS one 7: e49301 10.1371/journal.pone.0049301 23145148 PMC3492265

[28] Sobol RW, Kartalou M, Almeida KH, Joyce DF, Engelward BP, et al (2003) Base excision repair intermediates induce p53-independent cytotoxic and genotoxic responses. J Biol Chem 278: 39951–39959. 12882965 10.1074/jbc.M306592200

[29] Roth RB, Samson LD (2002) 3-Methyladenine DNA glycosylase-deficient Aag null mice display unexpected bone marrow alkylation resistance. Cancer Res 62: 656–660. 11830515

[30] Horton JK, Stefanick DF, Gassman NR, Williams JG, Gabel SA, et al (2013) Preventing oxidation of cellular XRCC1 affects PARP-mediated DNA damage responses. DNA Repair (Amst) 12: 774–785. 10.1016/j.dnarep.2013.06.004 23871146 PMC3924596

[31] Masaoka A, Gassman NR, Horton JK, Kedar PS, Witt KL, et al (2013) Interaction between DNA Polymerase β and BRCA1. PloS one 8: e66801 23826138 10.1371/journal.pone.0066801 PMC3694962

[32] Allard P, Colaiácovo MP (2011) Mechanistic insights into the action of Bisphenol A on the germline using C. elegans. Cell Cycle 10: 183–184. 21228622 10.4161/cc.10.2.14478

[33] Fernandez SV, Huang Y, Snider KE, Zhou Y, Pogash TJ, et al (2012) Expression and DNA methylation changes in human breast epithelial cells after bisphenol A exposure. Int J Oncol 41: 369–377. 10.3892/ijo.2012.1444 22576693 PMC3466112

[34] Yin R, Gu L, Li M, Jiang C, Cao T, et al (2014) Gene expression profiling analysis of bisphenol A-induced perturbation in biological processes in ER-negative HEK293 cells. PloS one 9: e98635 10.1371/journal.pone.0098635 24901218 PMC4047077

[35] Chan MK, Ocampo-Hafalla MT, Vartanian V, Jaruga P, Kirkali G, et al (2009) Targeted deletion of the genes encoding NTH1 and NEIL1 DNA N-glycosylases reveals the existence of novel carcinogenic oxidative damage to DNA. DNA Repair (Amst) 8: 786–794. 10.1016/j.dnarep.2009.03.001 19346169 PMC4894318

[36] Dizdaroglu M, Jaruga P (2011) Oxidatively Induced DNA Damage and Cancer. J Mol Biomark Diagn S2:002.

[37] Jaruga P, Xiao Y, Vartanian V, Lloyd RS, Dizdaroglu M (2010) Evidence for the involvement of DNA repair enzyme NEIL1 in nucleotide excision repair of (5′R)- and (5′S)-8,5′-cyclo-2′-deoxyadenosines. Biochemistry 49: 1053–1055. 10.1021/bi902161f 20067321 PMC2817919

[38] Audebert M, Dolo L, Perdu E, Cravedi JP, Zalko D (2011) Use of the γH2AX assay for assessing the genotoxicity of bisphenol A and bisphenol F in human cell lines. Arch Toxicol 85: 1463–1473. 10.1007/s00204-011-0721-2 21656223

[39] Lan L, Nakajima S, Oohata Y, Takao M, Okano S, et al (2004) In situ analysis of repair processes for oxidative DNA damage in mammalian cells. Proc Natl Acad Sci U S A 101: 13738–13743. 15365186 10.1073/pnas.0406048101 PMC518826

[40] Masaoka A, Gassman NR, Kedar PS, Prasad R, Hou EW, et al (2012) HMGN1 protein regulates poly(ADP-ribose) polymerase-1 (PARP-1) self-PARylation in mouse fibroblasts. J Biol Chem 287: 27648–27658. 10.1074/jbc.M112.370759 22736760 PMC3431713

[41] Dobrzynska MM, Radzikowska J (2013) Genotoxicity and reproductive toxicity of bisphenol A and X-ray/bisphenol A combination in male mice. Drug Chem Toxicol 36: 19–26. 10.3109/01480545.2011.644561 22263531

