# Supplementary figures and images for: Bisphenol A Promotes Cell Survival Following Oxidative DNA Damage in Mouse Fibroblasts
Source: PLoS One. 2015 Feb 18;10(2):e0118819. doi: 10.1371/journal.pone.0118819 (PMC4334494; doi:10.1371/journal.pone.0118819)

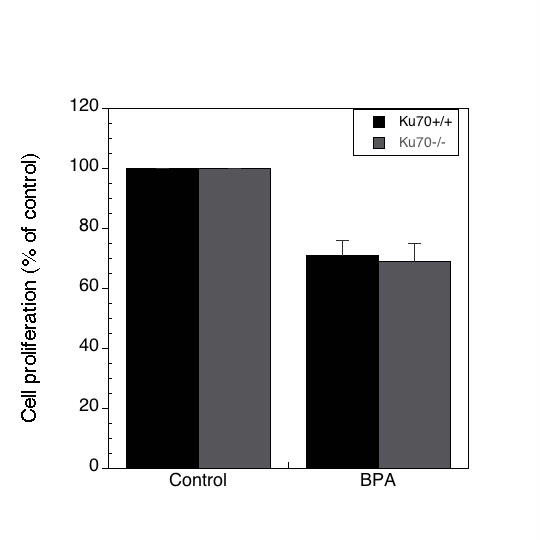

Supplement: S1 Fig — Cells were exposed to 150 μM BPA for 24 h as described in Material and Methods. Cells were then washed, normal growth medium was replaced, and cell survival was evaluated by CellTiter 96 AQueous One Solution Cell Proliferation Assay (Promega). Values shown are the percent change in cell number compared with control given as the mean ± SD of three independent experiments. (TIF) [file pone.0118819.s001.tif]

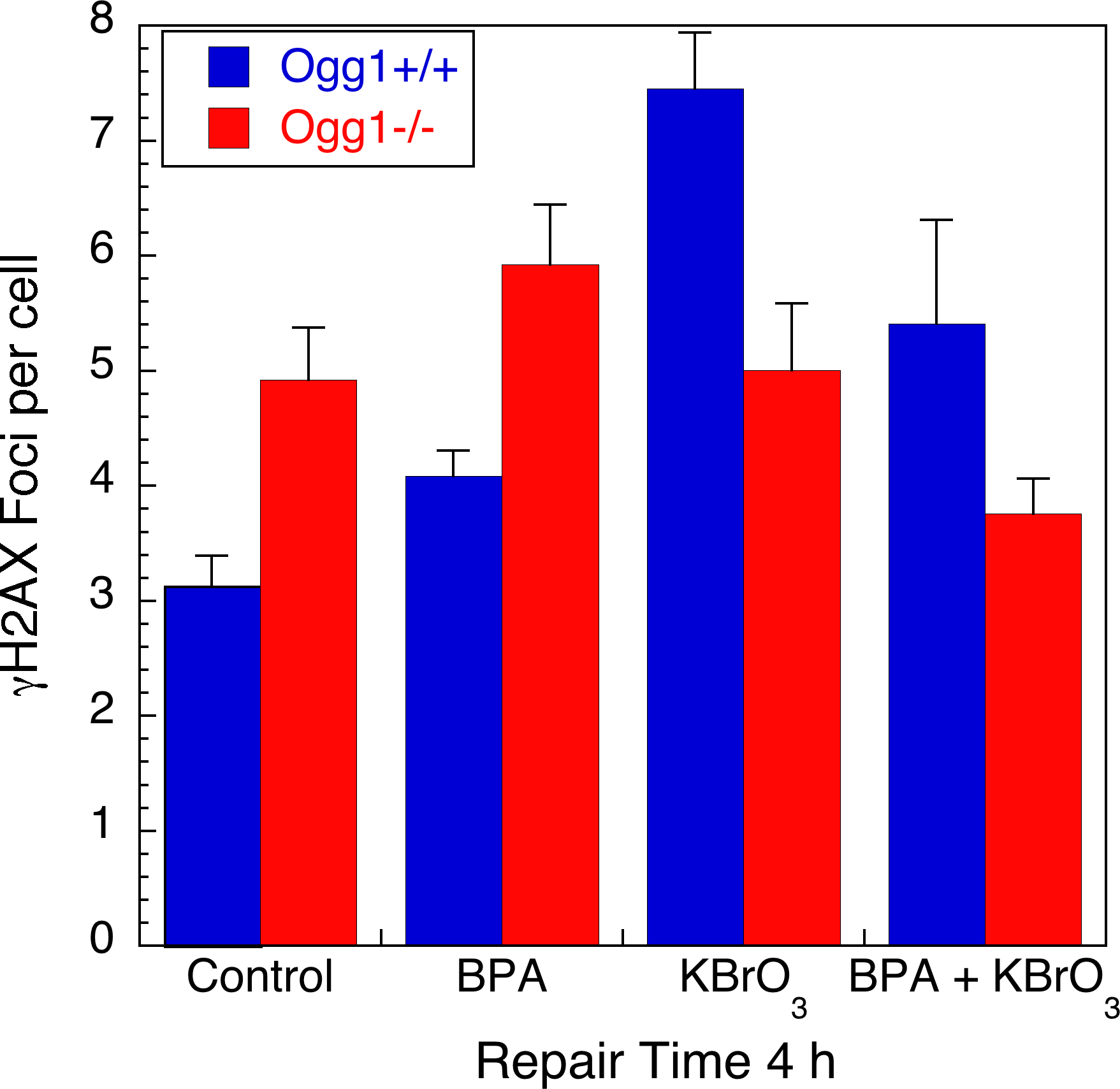

Supplement: S2 Fig — γH2AX foci were measured at 4 h after exposure to BPA alone, KBrO3 alone, and after co-exposure (see Material and Methods). Each bar represents mean ± SEM of three independent experiments. (TIF) [file pone.0118819.s002.tif]
