# Supplementary material for: Bisphenol A Promotes Cell Survival Following Oxidative DNA Damage in Mouse Fibroblasts
Source: PLoS One. 2015 Feb 18;10(2):e0118819. doi: 10.1371/journal.pone.0118819 (PMC4334494; doi:10.1371/journal.pone.0118819)
Supplement: S1 Table — (PDF) [file pone.0118819.s003.pdf]

Table S1. Levels of modified DNA bases in genomic DNA of Ku70

	DNA lesion/ 10^6 DNA bases (mean ± SD, n ≥3)				
	Ku70+/+				
	5-OH-Cyt	Thymine Glycol	FapyAde	FapyGua	8-oxoGua
Control	1.70	3.25± 1.26	1.86± 0.87	2.11 ± 0.73	1.48 ± 0.64
BPA	1.63	4.57± 2.08	1.82± 0.76	2.86 ± 1.27	0.91 ± 0.16
$KBrO_3$	0.91± 0.11	2.76± 0.93	1.87± 0.33	2.86 ± 0.81	0.83 ± 0.15
BPA + $KBrO_3$	2.61± 2.05	1.93	2.02± 0.78	3.54 ± 0.55*	2.63 ± 1.65
